# Development and Characterization of an Antibody-Labeled Super-Paramagnetic Iron Oxide Contrast Agent Targeting Prostate Cancer Cells for Magnetic Resonance Imaging

**DOI:** 10.1371/journal.pone.0097220

**Published:** 2014-05-12

**Authors:** David Bates, Suraj Abraham, Michael Campbell, Ingeborg Zehbe, Laura Curiel

**Affiliations:** 1 Thunder Bay Regional Research Institute, Ontario, Canada; 2 Department of Biology, Lakehead University, Thunder Bay, Ontario, Canada; 3 Department of Chemistry, Lakehead University, Thunder Bay, Ontario, Canada; 4 Department of Physics, Lakehead University, Thunder Bay, Ontario, Canada; San Raffaele Scientific Institute, Italy

## Abstract

In this study we developed, characterized and validated *in vitro* a functional superparagmagnetic iron-oxide based magnetic resonance contrast agent by conjugating a commercially available iron oxide nanoparticle, Molday ION Rhodamine-B Carboxyl (MIRB), with a deimmunized mouse monoclonal antibody (muJ591) targeting prostate-specific membrane antigen (PSMA). This functional contrast agent is intended for the specific and non-invasive detection of prostate cancer cells that are PSMA positive, a marker implicated in prostate tumor progression and metastasis. The two-step carbodiimide reaction used to conjugate the antibody to the nanoparticle was efficient and we obtained an elemental iron content of 1958±611 per antibody. Immunofluorescence microscopy and flow cytometry showed that the conjugated muJ591:MIRB complex specifically binds to PSMA-positive (LNCaP) cells. The muJ591:MIRB complex reduced cell adhesion and cell proliferation on LNCaP cells and caused apoptosis as tested by Annexin V assay, suggesting anti-tumorigenic characteristics. Measurements of the T2 relaxation time of the muJ591:MIRB complex using a 400 MHz Innova NMR and a multi-echo spin-echo sequence on a 3T MRI (Achieva, Philips) showed a significant T2 relaxation time reduction for the muJ591:MIRB complex, with a reduced T2 relaxation time as a function of the iron concentration. PSMA-positive cells treated with muJ591:MIRB showed a significantly shorter T2 relaxation time as obtained using a 3T MRI scanner. The reduction in T2 relaxation time for muJ591:MIRB, combined with its specificity against PSMA+LNCaP cells, suggest its potential as a biologically-specific MR contrast agent.

## Introduction

In the last 10 years, the incidence of prostate cancer has steadily risen, remaining the second most common cancer diagnosed in men worldwide. In Canada alone, it accounts for roughly 27% of newly diagnosed cancers in 2012 [1]. Prostate tumour growth and spread are often very slow and remain undetected at early stages of the cancer. Current detection and treatment planning heavily relies on the use of prostate specific antigen (PSA)-expressing cells. However, the low specificity of this test has led to overtreatment of early and less aggressive cancer and under-treatment of indolent but aggressive cancer, leading to high morbidity [2]. In addition, current treatments have high morbidity and possible post-treatment relapses, which compromise the patient's quality of life and survival [3], [4].

Studies have suggested that, by specifically targeting prostate specific membrane antigen (PSMA) expressing cells, both localized and aggressive conditions can be treated [3], [5]–[17]. PSMA is a highly characterized prostate cancer biomarker localized to the prostate cancer cell membrane, suggesting its usefulness for *in vivo* prostate cancer specific targeting strategies [8], [9], [13]–[17]. The PSMA gene has been cloned, sequenced, and mapped to chromosome 11q14, and it is expressed in a high proportion of malignant prostate epithelial cells, but not in the normal vascular endothelium [6]. In fact, PSMA is the single most well-established highly-restricted prostate epithelial cell membrane antigen, whereas PSA and prostatic acid phosphatase are secretory proteins [9], [17]. Immunotherapeutic and detection approaches that use anti-PSMA antibodies have been suggested as excellent tools for both detection and treatment of prostate cancer [8], [9], [13].

PSMA receptors are localized primarily to the apical plasma membrane of prostate epithelium and play an integral role in the progression of prostate malignancies, probably by promoting anti-apoptotic signaling, ensuring cell resilience and promoting cell proliferation [6], [18]. Targeting this receptor with the anti-PSMA J591 antibody has proven to be an effective detection and therapeutic tool for prostate cancer [10]–[12]. In order to be clinically applicable, the mouse monoclonal (mAb) anti-PSMA receptor is de-immunized by replacement of murine immunoglobulin sequences with human immunoglobulin sequences, resulting in a non-immunogenic, humanized antibody, huJ591 [9], [13]–[15]. The huJ591 mAb has been extensively used in phase I clinical trials, where it has been demonstrated to be well-tolerated without adverse host immune response [9], [14], [15].

Endorectal magnetic resonance imaging (MRI) is becoming common part of the local work-up for prostate cancer. Dynamic studies using gadolinium-DTPA contrast agents have demonstrated useful enhancement of tumor images [19]–[27]. Non-invasive detection of PSMA-expressing cells could ideally be performed by functional contrast-enhanced MRI techniques. Contrast agents enhance the contrast of tissues by altering relaxation times of tissues to improve the visibility of structures via MRI. Such detection methods require the development of functional MRI contrast agents, for instance linking anti-PSMA antibody to MRI contrast agents. Molday ION Rhodamine-B Carboxyl (MIRB) is a commercially available iron-oxide based nanoparticle that reduces T2 relaxation time of absorbing tissues and thus be detected as a loss in signal on MR images [28], [29]. The iron oxide core of the MIRB nanoparticle can be chemically activated to react with the amino terminal of proteins or antibodies to form a peptide linkage, yielding an antibody/protein:MIRB conjugate complex. A recent study demonstrated that MIRB can be used to label stem cells, cancer cells and immune cells preserving high cell viabilities and detectable by MRI [28]. MIRB's ease of conjugation with protein or antibody, its high loading concentration of iron for MR imaging, and good cell tolerability make it a good candidate for a functional antibody-labelled MR contrast agent.

In this study, we developed a functional super-paramagnetic iron oxide (SPIO)-based MR contrast agent that specifically attaches to PSMA receptors for MR imaging of prostate cancer cells. We present a method to develop this functional MR contrast agent using a commercially available SPIO, Molday ION Rhodamine-B Carboxyl (MIRB), conjugated to the anti-PSMA receptor antibody J591. We characterized and validated this antibody:SPIO-nanoparticle complex for specific detection of PSMA positive prostate cancer tumor cells using immunofluorescence, flow cytometry and 3T clinical MRI. Our new contrast agent can be used for the specific and non-invasive detection of prostate cancer cells with PSMA, a marker implicated in prostate tumor progression and metastasis [10]–[17], [30], [31].

## Materials and Methods

### Antibodies, MR contrast agents and reagents

Murine J591 mAb, at a concentration of 5 mg/mL and with known specificity for PSMA, was procured from Dr. Neil H Bander's laboratory (Laboratory of Urological Oncology, Weill-Cornell Medical College, New York, NY, USA) through an institutional material transfer agreement. The isotype control antibody for synthesis and cell culture experiments was a murine IgG1 (Monoclonal Mouse IgG1 Clone# 11711; Cat #: MAB002, R&D systems, Minneapolis, USA) at a concentration of 5 mg/mL in 1× phosphate buffered saline without calcium or magnesium ions (Cat #: SH30028.02, Hyclone Inc, Logan, UT, USA), as per manufacturer's recommendation.

Molday ION Rhodamine Carboxyl (MIRB, Cat #: CL-50Q02-6C-50, BioPAL, Inc, Worcester, MA, USA) was used to prepare the antibody:SPIO complexes. All the reagents required for the conjugation reactions and analysis, including *N*-(3-dimethylaminopropyl)-*N*′-ethylcarbodiimide hydrochloride (EDC), N-hydroxysuccinimide (NHS), 2-(4-Morpholino)ethanesulphonic acid hydrate (MES), sodium bicarbonate, HPLC-grade water (ChromoSolv Water), chloroform, acetonitrile, and deuterium oxide, were purchased from Sigma Aldrich, Oakville, ON, Canada, unless otherwise stated.

### Antibody labelling

Antibodies were conjugated to MIRB based on a modification of the manufacturer's method (Cat # CL-50Q02-6C-50, BioPAL, Inc, Worcester, MA, USA). Briefly, MIRB (0.2 mg of iron) was added to 100 µL of muJ591 or muIgG at 1 mg/mL in 1× PBS buffer (pH 7.65) in sterile 1.5 mL microfuge tubes. The antibody with MIRB solution was lightly vortexed prior to the addition of 100 µL of 0.1 M sodium bicarbonate buffer (pH 8.0), ice incubated for 15 min with occasional vortexing, then kept 24 h at 4°C in a rotator mixer. The overnight incubated antibody:MIRB were mixed with 50 µL of 2 mg/mL EDC solution in deionized water plus 100 µL of MES buffer (pH 5.6) and kept at 37°C for 5 min with occasional vortexing, prior to addition of 50 µL of 2 mg/mL NHS and incubation at 37°C for further 15 min, with occasional mixing. The solution was then cooled on ice for 15 min and kept at 4°C for 48 h in a rotator mixer. After 48 h the mixture was transferred to a sterile 15 mL conical tube and rinsed twice with 500 µL 1× PBS buffer to extract out the antibody:MIRB conjugates attached to the tube wall. In order to remove any unbound antibodies, the reaction mixture was transferred to a fresh sterile 15 mL conical tube containing 50 µL of 2 mg/mL NHS plus 150 µL of 0.25 M MES buffer and vortexed. This tube was kept overnight at 4°C in a rotator mixer, washed with an equal volume of 0.1 M Sodium bicarbonate buffer (pH 8.0) and centrifuged using 10 K Amicon Ultra Centrifugal Filters (Cat #: UFC201024, Millipore Ireland Ltd, Cork, Ireland), based on the manufacturer's protocol for ultrafiltration. The solution retained in the filtration column, containing the remaining antibody:MIRB conjugates, was washed with 0.01 M sodium bicarbonate (pH 7.65) using the buffer exchange steps described in the manufacturer's protocol. Each of the eluants was analyzed for both antibody and iron contents using the Bradford protein assay kit and inductive coupled plasma atomic emission spectroscopy (ICP-AES) based assay, respectively. The total protein concentration of the conjugates was determined by Bradford protein assay using gradient concentration of BSA and human γ-globulin as standards for indirect estimation of antibody content.

### Elemental iron content analysis

ICP-AES was used to determine the elemental iron contents of the antibody:MIRB conjugates. Antibody:MIRB conjugates were acid digested using an equal volume of 70% nitric acid in deionized LC-MS grade water, in a final sample volume of 6 mL; a buffer only (negative control) was similarly digested. All the samples were run on the Varian Vista Pro CCD simultaneous ICP-OES instrument (Varian Inc, Palo Alto, CA, USA).

### Scanning Electron Microscopy (SEM) and Energy Dispersive X-ray (EDX) spectroscopy

The complex muJ591:MIRB was immobilized on a Nucleopore membrane (Cat #: 800280, Whatman, Florham Park, NJ, USA) and heated at 57°C in a hot air oven for 25 min to remove moisture. The immoblized complex was then mounted and sputter-coated with carbon. SEM images were acquired at 5000 and 10,000× magnification using a Hitachi Su-70 Schotty Field Emission SEM (Hitachi High Technologies America Inc, Dallas, TX, USA) with a resolution of 1.5 nm at 1 kV. The same system was used to perform Energy Dispersive X-ray (EDX) spectroscopy at 5000× magnification to confirm the presence of organic matter and SPIO nanoparticles.

### Particle-size analysis

Particle size of muJ591:MIRB conjugate was determined by dynamic light scattering. The muJ591:MIRB conjugate was dispersed in 0.9% saline at a protein concentration of 0.01% w/v. Measurements were carried out using a Zetasizer Nano ZS instrument (Malvern Instruments, Worchestershire, UK). The particle size results were reported as the harmonic intensity averaged particle diameter (or Z-average) and they are expressed in nanometers.

### Nuclear Magnetic Resonance (NMR) analysis

To determine whether the muJ591:MIRB conjugate generated from the labelling reactions retained the super-paramagnetic characteristics required of an MR contrast agent, we measured the T2 relaxation time in NMR. Samples with equal total protein concentration (0.04 µg/µL) of muIgG:MIRB and muJ591:MIRB complexes were separately prepared in 600 µL of deuterium oxide and transferred into Wilmad NMR tubes (Cat#: Z272019, Sigma Aldrich, St. Louis, MO, USA). T2 relaxation times were obtained after optimizing the magnetic field of the Innova Unity 500 NMR machine and samples were run as per the manufacturer's recommendation.

### Fluorimetric analysis

To test whether the conjugation reaction caused fluorescence enhancement or quenching of the Rhodamine-B fluorophore on the MIRB conjugates we performed a fluorimetric analysis. MIRB nanoparticles and muJ591:MIRB complexes samples with an equal iron concentration were analyzed for fluorescence intensity and wavelength at peak intensity (λ_max_) using a Synergy4 fluorimetric plate reader (BioTek, Winooski,VT, USA). The peak λ_max_ and λ_max_ shift were determined by plotting the fluorescence intensity values at different excitation and emission wavelengths.

### Cell culture

PSMA-positive LNCaP cells (CRL-1740) and PSMA-negative DU145 cells (HTB-81) were procured from American Type Cell Culture, Manassas, VA, USA. All cells were grown in a 37°C, 5% CO_2_ incubator using RPMI-1640 media supplemented with 10% fetal bovine serum (FBS) and 1% antibiotics/antimycotics. The cells were grown to 80% confluence in 75 cm^2^ vented cap, canted neck falcon flasks (Cat #: 353136, BD Biosciences, Franklin Lakes, NJ, USA) with media replenished every three days.

### Antibody:MIRB complex specificity for PSMA

Cells were seeded at an initial concentration of 1000 (for DU145) and 2000 cells (for LNCaP) per well in 96 well plates and grown for 3 days before performing the experiments. Immunofluorescence studies were then conducted on both LNCaP and DU145 after treatment with 0.1 µg/µL muJ591:MIRB for 1 h with or without addition of secondary antibody conjugated to AlexaFluor-488 fluorophore. Then, cells were incubated with warm 0.5% BSA in RPMI media to encourage cells to internalize the antibody. The cells were then visualized at different time points (15 min, 30 min, 1 h, 1.5 h, 2 h, 4 h, 6 h and 12 h) under a standard inverted fluorescence microscope (Axiovert 200, Carl Zeiss. Gottingen, Germany) attached to a closed circuit camera (12-bit, Q-Imaging, Surrey, Canada).

A flow cytometric assay was performed based on a modification of the method described by Peldschus K et al (2010) [32]. Cells were detached using trypsin-EDTA (Invitrogen, Carlsbad, CA) which was inactivated through washing with 10% FBS RPMI-1650. Cells were then washed twice using serum-free RPMI-1640, prior to the addition of 250 µL muIgG, muIgG:MIRB, muJ591, muJ591:MIRB (protein concentration of 0.05 µg/µL) followed by 1 hour of incubation on ice. The untreated controls were incubated in an equivalent volume of serum-free media. Cell bound antibody conjugates were marked for detection using Phycoerthyrin-conjugated goat anti-mouse IgG (Cat #550589, BD Pharmingen). Flow cytometry was then performed using a FACSCalibur device (Becton Dickson, Franklin Lakes, NJ, USA). Fluorescence measurement were given as geometric means and medians of 10,000 events and plotted as diagrams. Experiments were done in triplicates and we compared the signal intensity shift between untreated cells and cells treated with muIgG, muIgG:MIRB, muJ591, or muJ591:MIRB.

### Cell adhesion

Cell adhesion after treatment of plates with muJ591 and muJ591:MIRB was evaluated. The muJ591:MIRB complex and muJ591 mAb was prepared in 1× phosphate buffered saline (PBS) at a concentration of 0.01 µg/mL. We then precoated 96-well plates with 100 µL of muJ591:MIRB complex and muJ591 mAb in quadruplicates, and incubated overnight at 4 °C for immobilization of the complex on the plate surface. After overnight incubation, we aspirated out the solution and blocked the wells with 1% BSA in 1× PBS for at least 10 minutes and aspirated out again. Non-treated wells were used as control. About 100,000 LNCaP or DU145 cells were then seeded in the precoated and control wells and incubated at 37°C, 5% CO_2_ for 40 minutes. Cells were finally washed with serum-free RPMI-1640 until cells in the uncoated wells stopped detaching. All cells were then fixed using ice-cold methanol and stained with 0.1% crystal violet. Filtered 2% SDS was added and the plates were incubated with agitation at room temperature for 30 minutes, after which time, absorbance readings at 550 nm were taken using the PowerWave XS plate reader (BioTek, Winooski,VT, USA). We compared the absorbance readings from the muJ591- and muJ591:MIRB-coated plates with respect to the untreated control wells to analyze adhesion.

### Cell proliferation and apoptosis

For cell proliferation assessment, a resazurin-based fluorimetric assay (Cat #AR002, R&D Systems, Minneapolis, MA, USA) was performed. After the 96 well plates were precoated with muJ591:MIRB or 1× phosphate buffered saline (control) and blocked with 1% BSA as described above, 1000 DU145 or 2000 LNCaP cells were seeded. The cells were grown until control wells reached 80% confluence. The LNCaP and DU145 cells were further treated with muJ591:MIRB complex (0.01 µg/mL) or serum free media (control) for 1 h, after which media was replaced with serum free media. The Resazurin solution was added, as recommended by the manufacturer's protocol, and plates were further incubated at 37°C, 5% CO_2_ for 2 hours followed by 20 min incubation with agitation in the dark at room temperature. The fluorescence intensities were then measured at 2, 6, 12 and 24 h post muJ591:MIRB treatment using the FLx800 plate reader at excitation of 540 nm and emission of 590 nm.

For determining cell apoptosis and death caused by the muJ591:MIRB complexes on LNCaP cells, an Annexin V-FITC assay was performed (Sigma Aldrich, St.Louis, MO, USA). Cells were dislodged from the flasks using 2 mM EDTA in 1× phosphate buffer saline, pelleted and resuspended in sterile, supplemented media, and then seeded in 6 well culture plates at a concentration of 10,000 cells per well. Once cells reached 80–90% confluence, muJ591:MIRB or control (muIgG:MIRB, 0.01 µg/mL and serum free media) treatment was added to the wells for 1 h followed by 3 washes in phosphate-buffered saline (PBS). Flow cytometric analysis was then performed based on method described by Richard et al (2010) [33].

### *In vitro* MR imaging

To validate we could detect changes on the T2 relaxation time with a clinical MR scanner we performed MR imaging of the conjugates first and then conjugate-treated cells. The muJ591:MIRB and muIgG:MIRB complexes, as well as muJ591, muIgG and MIRB alone were suspended on PBS at different concentrations (0.28, 0.14, 0.07, 0.04, 0.02 and 0.01 µg/µL protein concentration for the antibodies; and 0.13, 0.06, 0.03, 0.02 µg/µL iron concentration for the MIRB). Then, 100 µL of serum free media were added to 96-well plates (n = 3) and placed on the MR scanner.

For *in vitro* cell MR imaging, both LNCaP and DU145 cells were plated, in triplicate, at concentrations of 10^5^ cells/well in 96 well plates and grown for 36–48 h. The cells were then treated with 100 µL of antibody:MIRB conjugates or non-conjugated antibodies for 1 h, rinsed 2× times with serum free media, 1× time with 1× Tris-EDTA buffered saline, and 1× times with 0.01 M Sodium bicarbonate buffer to remove unbound conjugates, and then resuspended in 100 µL serum free media and placed on the MR scanner. Different concentrations of muJ591:MIRB, muJ591, muIgG:MIRB and muIgG (0.28, 0.14, 0.07, 0.04, 0.02 and 0.01 µg/µL protein concentration) were used.

To determine the limit of detection LNCaP cells were plated in 96 well plates, in triplicate, at concentrations of 10^5^, 7.5^5^, 5^5^, 2.5^5^ and 1.25^5^ cells/well and grown for 48 h. The cells were then treated with 100 µL of muJ591:MIRB for 1 h, rinsed 2× times with serum free media, 1× time with 1× Tris-EDTA buffered saline, and 1× times with 0.01 M Sodium bicarbonate buffer to remove unbound conjugates, resuspended in 100 µL serum free media and placed on the MR scanner.

A clinical 3T MRI scanner (Achieva 3.0T TX, Philips, Best, The Netherlands) with a small surface Flex coil was used. Images were acquired with a multi-echo spin echo sequence with 64 echo times spaced by 5.8 ms (TE/TR = N*5.8/1245 ms, FOV = 160 mm×160 mm, 1.5 mm slice thickness, 184×184 acquisition matrix, 384×384 reconstruction matrix, flip angle 90°, ETL = 32, 1 NEX, where N is the number of echo from 1 to 64). The intensity as a function of echo time was fitted to:
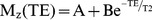
(1)


Where M_Z_ is the intensity at echo time TE; A, B and T2 are the fitting parameters [34]. A parametric imaging of the T2 relaxation time was obtained for all images of antibody samples and antibody-treated cells. The average T2 relaxation time for each antibody and live cell sample was obtained from the T2 values of each pixel in these parametric images.

### Statistical analysis

All statistical analyses were performed using Graphpad Prism software (GraphPad Software Inc, La Jolla, CA, USA). All data were tested for normality and homogeneity of variances using a Shapiro-Wilks test and a Bartlett's test, respectively, before choosing a suitable parametric or non-parametric statistical test. A resulting p-value of less than 0.05 was considered to be significant. Parametric data sets were analysed using Student's t-tests or one-way ANOVAs, followed by an appropriate post-hoc such as a Tukey HSD. Non-parametric data sets were analysed using one-way Kruskal Wallis ANOVA followed by a Nemenyi's post hoc, and Friedman's tests followed by a pair-wise comparison using a Wilcoxon signed rank test with a Bonferroni adjustment.

## Results

### Antibody:MIRB complex molecular characterization

**Figure 1:**
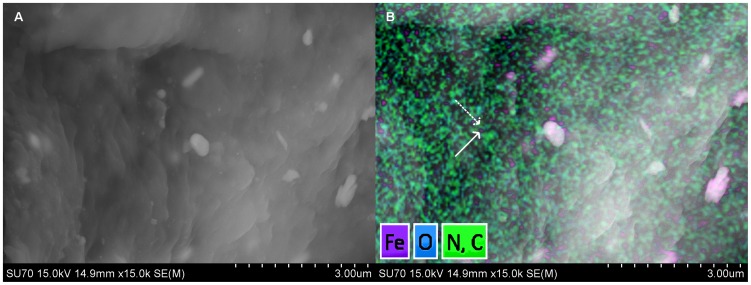
muJ591:MIRB characterization. A. Scanning Electron Micrograph (SEM) of immobilized muJ591:MIRB complexes and B. Energy Dispersive X-ray (EDX) mapping for Fe, C, O and N superimposed on the SEM. The presence of MIRB nanoparticles can be observed as iron signal (solid arrow) and the antibody as organic matter signal (dashed arrow). The large white structures are salts from the dried buffer.

ICP-AES was used to estimate the amount of elemental iron present in each of the conjugated muIgG:MIRB and muJ591:MIRB complexes. We observed that muJ591:MIRB complexes had 1958±611 (n = 8) elemental iron per antibody while muIgG:MIRB complexes had 2906±631 (n = 8) elemental iron per antibody. The iron nanoparticles presence on the muJ591:MIRB complex was further confirmed by Energy Dispersive X-ray spectroscopy (Figure 1). The presence of the iron was also confirmed by a fast T2 relaxation time obtained by NMR analysis at 400 MHz, which was 12.9±0.7 ms for the muJ591:MIRB complex and 10.2±0.6 for the muIgG:MIRB complex.

The presence of the muJ591:MIRB complex immobilized on salt crystals from the buffer can be observed in the superposed EDX-SEM images (Figure 1). EDX mapping showed the presence of carbon, nitrogen and hydrogen suggesting the presence of organic components (antibody) as well as iron from the MIRB nanoparticle.

The average particle sizes for the muJ591:MIRB conjugates were 34.75±14.41 nm with a polydispersity index (PdI) value of 0.23±0.01. For comparison, we performed particle size measurements for the MIRB nanoparticles alone and obtained 34.64±10.83 nm with a PdI of 0.25±0.02 which are within the values reported by the manufacturer (35 nm).

### Antibody:MIRB complex specificity

**Figure 2:**
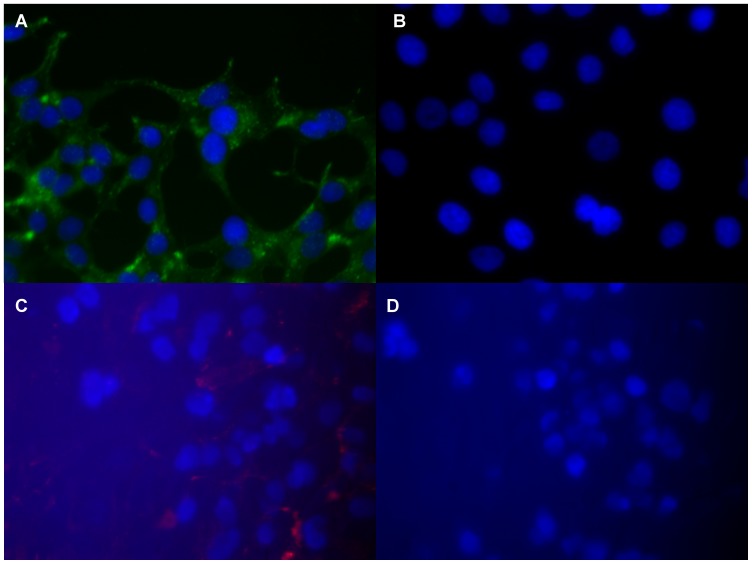
Immunocytochemistry. Fluorescence microscopy-based detection of PSMA-positive prostate cancer cell line with muJ591:MIRB complex: Secondary antibody staining conjugated to AlexaFluor-488 shows the binding of the J591 antibody in (A) LNCaP and whereas (B) DU145 control cells have no binding; and (C) Rhodamine-B fluorophore was observed on LNCaP cells (PSMA-positive) and not on (D) DU145 cells (PSMA-negative).

Immunofluorescence microscopy was used to determine whether PSMA positive LNCaP cells were specifically detected by the muJ591:MIRB complex. Figure 2 shows the 2 h post-treatment time-point for both LNCaP and DU145 cells treated with muJ591:MIRB complex. AlexaFluor-488 staining allowed for localization of the antibody within sub-cellular and extracellular regions of PSMA-expressing LNCaP cells (Figure 2A). The Rhodamine-B fluorophore (red) that is attached to MIRB was specifically detected in PSMA-expressing LNCaP cells (Figure 2C). No red or green fluorescent signals were observed in DU145 control cell line (Figure 2B and 2D) suggesting high specificity of muJ591:MIRB complex in detecting PSMA-positive prostate cancer cells.

**Figure 3:**
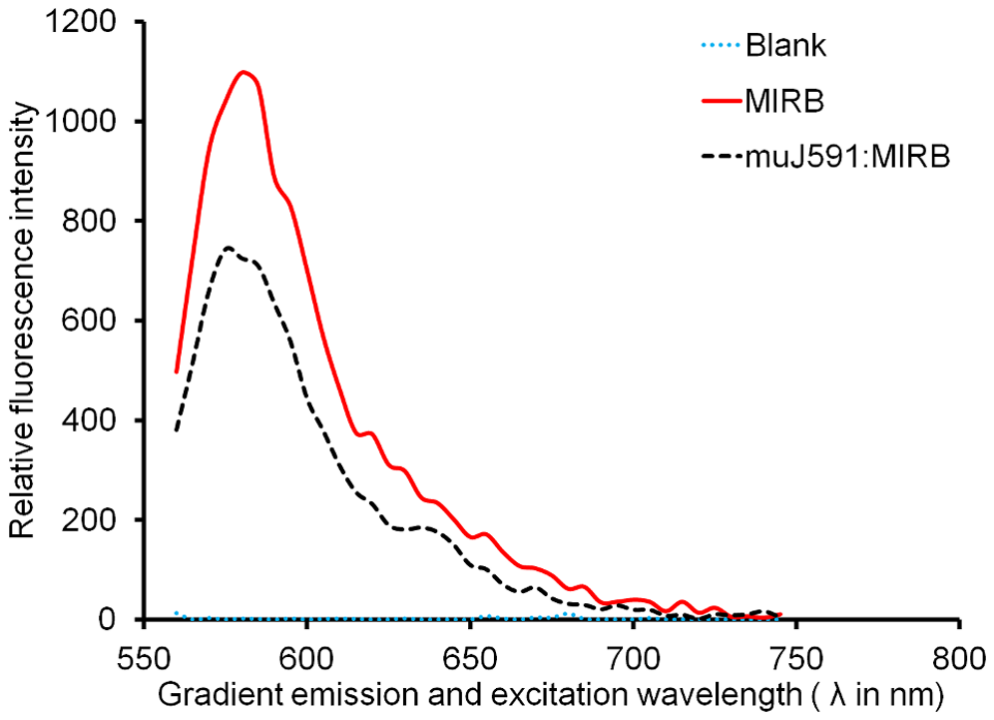
Fluorimetric changes. Effect of antibody conjugation on the relative fluorescence intensity of Molday ION Rhodamine-B complex (n = 3).

Fluorescence quenching and emission enhancement have been reported for metal-fluorophore conjugate systems [35]. We determined that fluorescent quenching occurred in our system by comparing the fluorescence intensity and peak λ_max_ of MIRB alone and muJ591:MIRB complex (Figure 3). The λ_max_ for MIRB alone was 575 nm, while for the muJ591:MIRB complex, the λ_max_ showed two distinct peaks between 560 to 575 nm. In addition, the fluorescence intensities between the peak λ_max_ values was 40±2% lower for the muJ591:MIRB complex compared to MIRB alone. This reduction in peak value and the shift in peak λ_max_ values for the Rhodamine-B fluorophore suggest that the conjugation may quench the fluorescence capacity of Rhodamine-B fluorophore for the complex.

**Figure 4:**
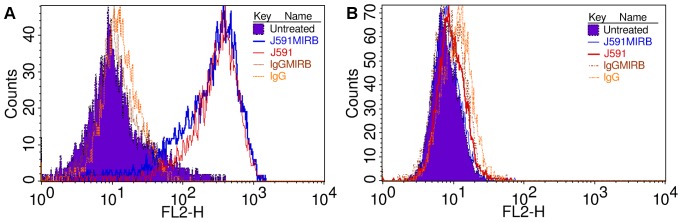
Flow cytometry. Specificity and uptake of muJ591:MIRB, muIgG:MIRB, muJ591 and muIgG of prostate cancer cells. A. LNCaP cells presented a shift that showed uptake of the muJ591 and of the conjugate muJ591:MIRB and no uptake for the controls muIgG antibody or muIgG:MIRB antibody conjugate. B. Control DU145 cells did not show uptake.

The specificity and uptake of muJ591:MIRB compared with the non conjugated muJ591 and control muIgG was examined on live prostate cancer cell lines using flow cytometry. LNCaP cells treated with muJ591 or muJ591:MIRB showed a significant fluorescence intensity shift on FL-2 compared with the untreated cells or MIRB-only treatment and they both had a similar shift (Figure 4A). The shift for muJ591 and muJ591:MIRB treatment of LNCaP cells show that there is specific and high uptake of the antibody and antibody-nanoparticle complex by the PSMA positive cells and that it does not change after the conjugation with the nanoparticle. Non-specific binding as tested by the muIgG and muIgG:MIRB was not observed (Table 1). Control DU145 cells devoid of PSMA did not show shift in intensity for any treatment (Figure 4B).

**Table 1:** Geometric mean and median of 10,000 events obtained by flow cytometry for different cell lines and treatment with the antibody and antibody:MIRB conjugates.

	LNCaP		DU145	
Treatment	Geometric mean	Median	Geometric mean	Median
muJ591:MIRB	259.12	352.27	9.45	8.90
muJ591	361.41	425.51	10.47	10.09
muIgG:MIRB	16.35	12.75	14.20	15.26
muIgG	15.58	13.58	15.94	16.70
Untreated	11.89	13.58	9.08	8.66

### Antibody:MIRB complex functionality

**Figure 5:**
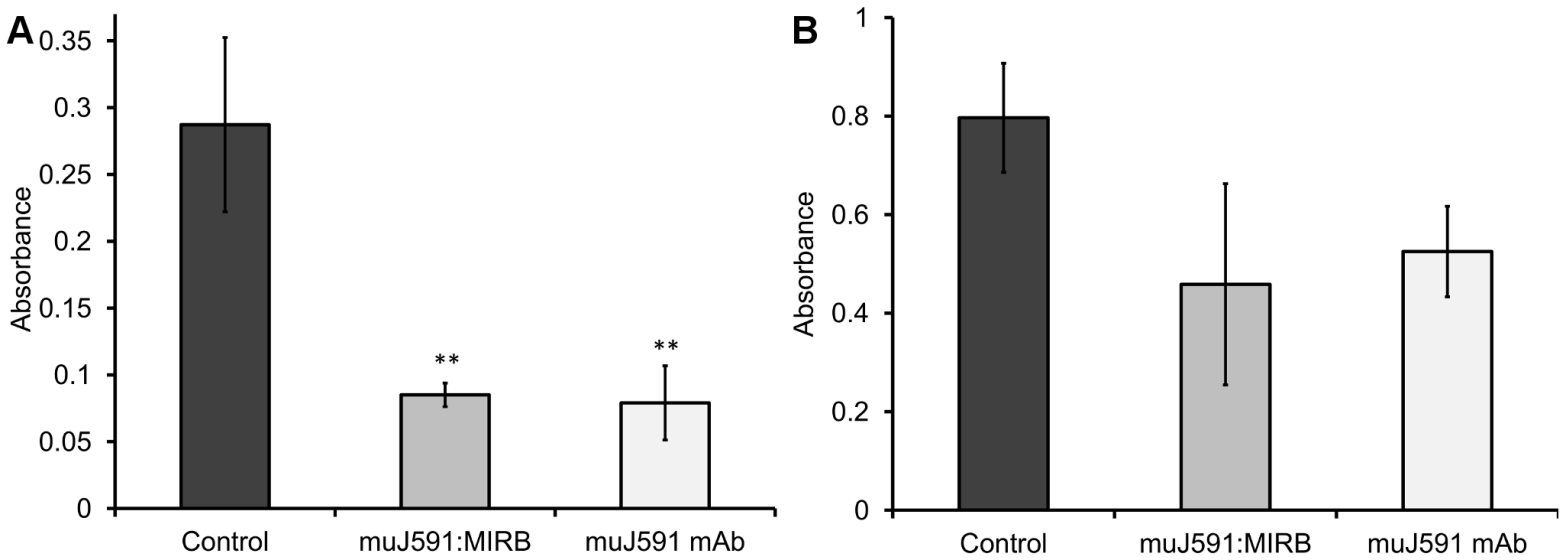
Cell adhesiveness. A. Significantly reduced adhesion on LNCaP cells (Student's t-test,** p<0.01, n = 4) was observed as a reduction on absorbance caused by both muJ591 mAb and muJ591:MIRB coating. B. Adhesion was also reduced by this coating on DU145 cells but the effect was not so pronounced.

We determined the potential anti-proliferative, pro-apoptotic and anti-tumorigenic effects of the muJ591:MIRB conjugated nanoparticles. On plates coated with both muJ591 and muJ591:MIRB the absorbance reading showed a reduced adhesiveness on LNCaP cells seeded after coating (Figure 5A, p<0.01). There was no difference on the effect on adhesiveness between the muJ591 antibody and the muJ591:MIRB complex (p = 0.7). The coating had also an effect on DU145 cells adhesiveness but it was less pronounced (Figure 5B). These results suggest that the effect of muJ591 antibody on cell adhesiveness remained unaffected by the antibody labelling with MIRB.

**Figure 6:**
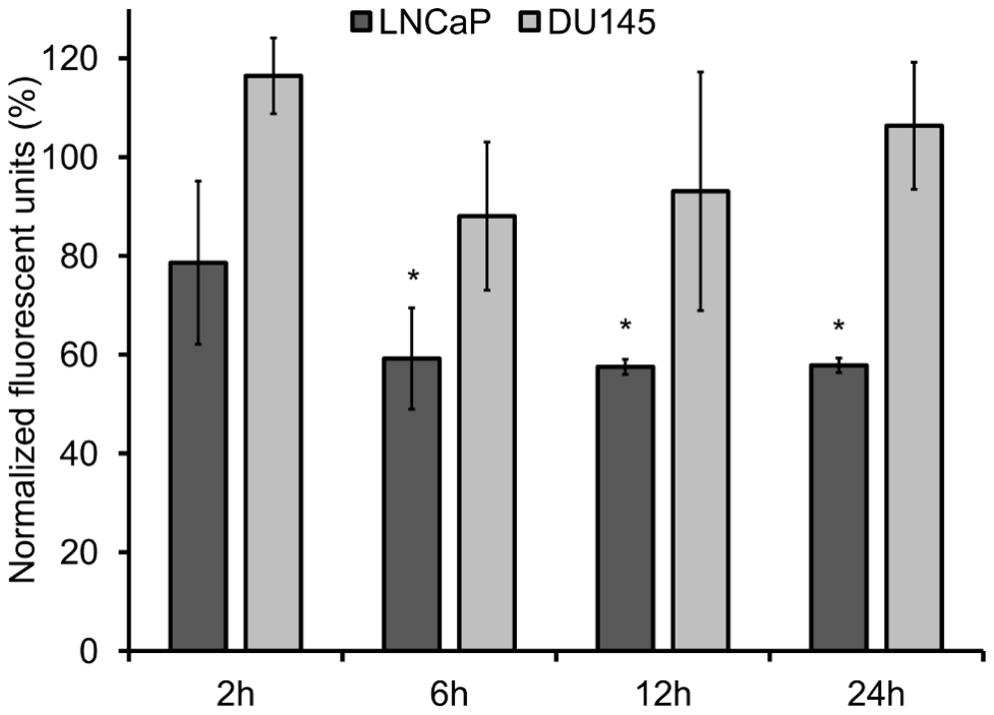
Cell proliferation. Effect on cell proliferation of LNCaP and DU145 prostate cancer cells following muJ591:MIRB treatment at different time-points obtained as the normalized resazurin fluorescent unit (RFU) value for treated cells over non-treated cells. LNCaP cells showed a significantly lower RFU than control vehicle-treated LNCaP cells starting at the 6 h time-point, while no significant changes were observed in muJ591:MIRB-treated DU145 cells. Student t test, *p<0.05 considered significant, n = 4.

Resazurin-based fluorometric assays performed at 2, 6, 12, and 24 h post-treatment determined the anti-proliferative effects of muJ591:MIRB on PSMA-positive LNCaP prostate cancer cells. A time-dependent decrease in cell proliferation for LNCaP cells and no change in PSMA-negative DU145 cells was observed (Figure 6). This suggests that muJ591:MIRB was highly specific in limiting the cell proliferation of the LNCaP cells.

**Figure 7:**
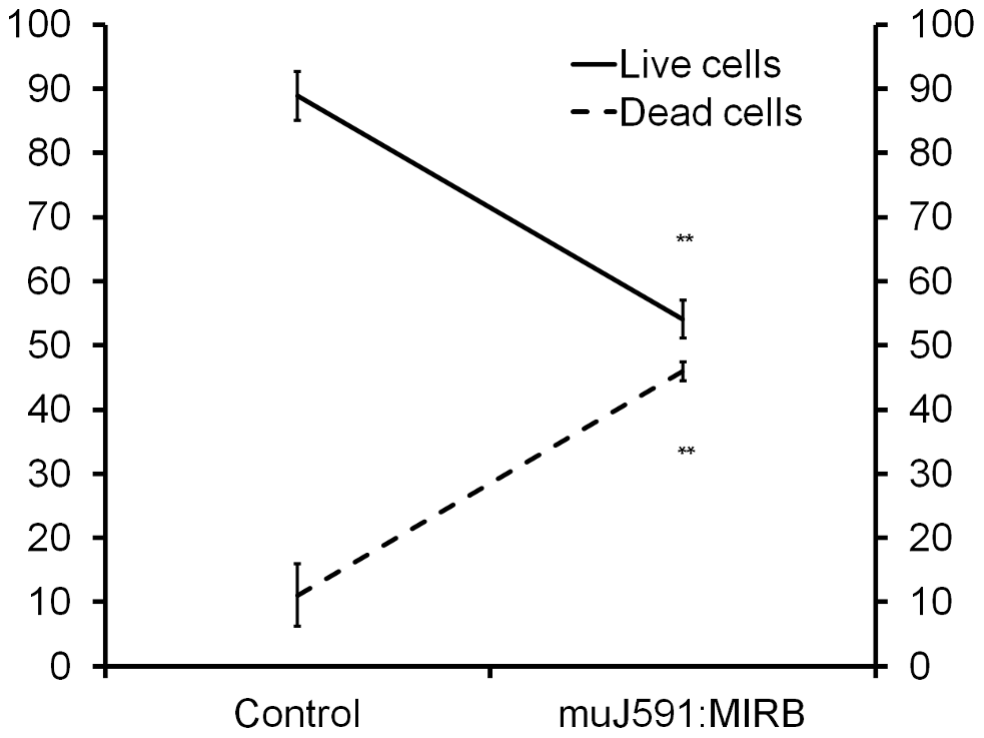
Cell death. Effect of muJ591:MIRB on the percentage of live over dead LNCaP cells (apoptotic and necrotic) calculated using Annexin V-FITC assay. One way ANOVA, ***p<0.0001 considered significant, n = 4.

There were significantly higher levels of Annexin V positive cells in muJ591:MIRB-treated LNCaP cells compared to the control (vehicle or muIgG:MIRB) treated LNCaP cells. Figure 7 shows the percentage of live and dead LNCaP cells as evaluated by Annexin V flow cytometry in the presence of muJ591:MIRB and control treatments; the number of live cells was significantly reduced while the number of dead cells (apoptotic and necrotic cells) was significantly higher.

### *In vitro* MR imaging

**Figure 8:**
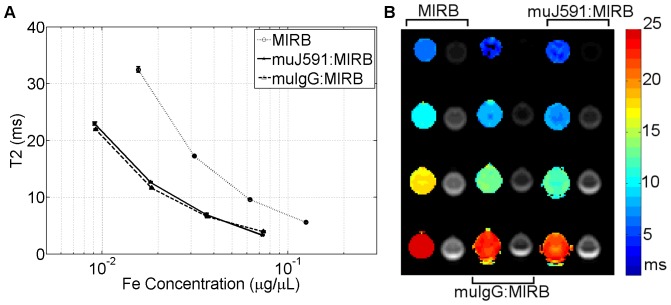
T2 relaxation time of conjugates. A. T2 relaxation time as a function of the iron concentration for the conjugates and free MIRB nanoparticles (n = 3 for each iron concentration); B. Corresponding MR parametric maps of T2 relaxation times (color) and intensity imaging (TE = 5.8 ms) of samples at different iron concentrations for MIRB alone, muIgG:MIRB and muJ591:MIRB conjugates.

The complex antibody:MIRB demonstrated faster T2 relaxation time with increasing iron concentration (Figure 8). For non-conjugated muJ591, the average T2 relaxation time over the sample well was 1669±59 ms and for non-conjugated muIgG antibody it was 1865±78 ms (n = 3). Both muJ591:MIRB and muIgG:MIRB had a shorter T2 relaxation time compared to non-conjugated antibody and the relaxation time became faster as a function of iron concentration. The changes in T2 relaxation time were consistent with the behaviour observed from the NMR measurements.

**Figure 9:**
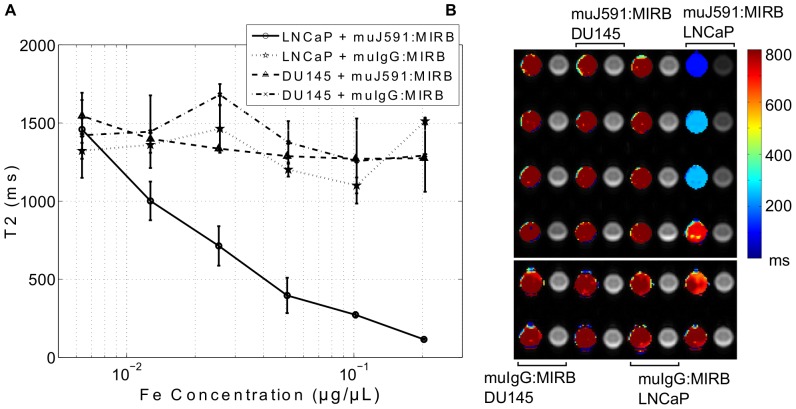
T2 relaxation time of treated cells. A. T2 relaxation time as a function of the iron concentration for PSMA-positive (LNCaP) and PSMA-negative (DU145) live prostate cancer cells treated with the antibody:MIRB conjugates (n = 3 for each concentration); B. Corresponding MR parametric maps of T2 relaxation times (color) and intensity imaging (TE = 5.8 ms) of samples treated with different antibody:MIRB concentrations (0.28, 0.14, 0.07, 0.04, 0.02, 0.01 µg/µL protein concentration equivalent to 0.2, 0.1, 0.05, 0.03, 0.012, 0.006 µg/µL in iron).

Live PSMA-positive (LNCaP) prostate cancer cells showed a reduced T2 relaxation time when treated with the muJ591:MIRB complex (Figure 9), and T2 was faster for higher concentrations of the antibody (equivalent to a higher concentration of iron). None of the PSMA-negative (DU145) cells showed a change in T2 regardless of the antibody/iron concentration. The muIgG:MIRB-treated LNCaP or DU145 cells did not show a change in T2 and they had an average T2 relaxation time of 1327±162 ms and 1412±202 ms respectively. This value was not statistically different from untreated cells, indicating no detectable unspecific binding of the control antibody to the cells. When LNCaP cells were treated with non-conjugated antibody, the T2 remained unaffected with an average of 1382±59 ms and 1548±37 ms for muJ591 and muIgG treatment, respectively. PSMA-negative DU145 cells treated with non-conjugated antibody did not show a change in their T2 relaxation time and registered an average of 1298±6 ms and 1527±27 ms for muJ591 and muIgG treatment, respectively. This confirmed that the muJ591:MIRB complex specifically detected PSMA-positive LNCaP cells using a clinical 3T MRI.

**Figure 10:**
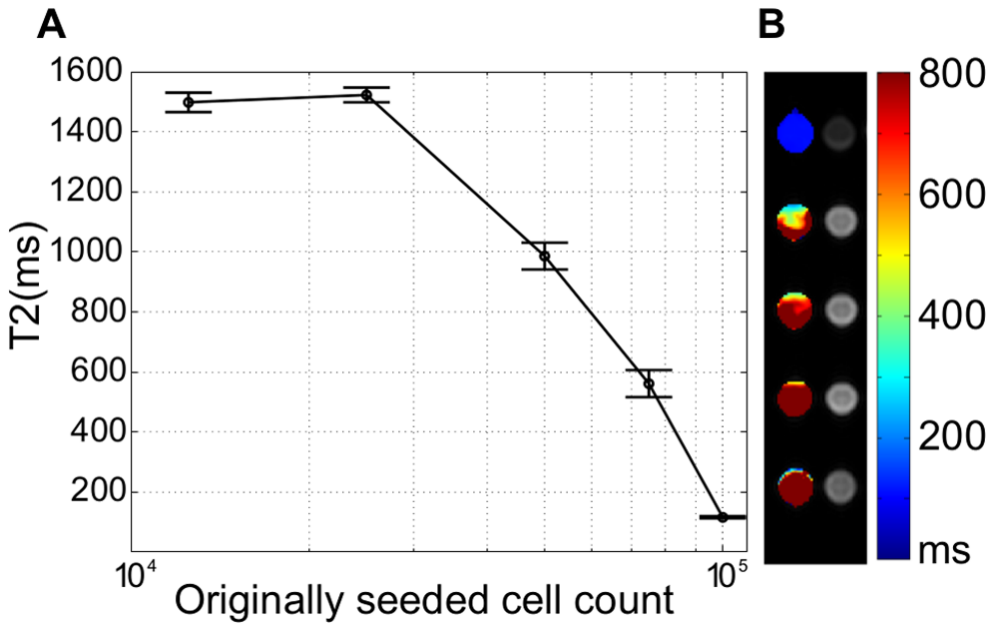
T2 relaxation time for different seeded counts of LNCaP treated cells. A. T2 relaxation time as a function of the originally seeded LNCaP cells (n = 3); B. Corresponding MR parametric maps of T2 relaxation times (color) and intensity imaging (TE = 5.8 ms) of samples. All cells were treated with muJ591:MIRB at 0.28 µg/µL protein concentration equivalent to 0.2 µg/µL in iron.

For live PSMA-positive (LNCaP) prostate cancer cells treated with muJ591:MIRB the T2 relaxation time became faster for higher seeded cell counts (Figure 10). No changes on T2 relaxation time compared to untreated LNCaP cells could be observed for 2.5^5^ or lower seeded cell counts, showing there is a limit on the detection provided by the MRI that depends on the cell population.

LNCaP and DU145 prostate cancer cells also showed a reduced T2 relaxation time when treated with the MIRB nanoparticle alone, with a faster T2 for higher iron concentrations. LNCaP cells treated with MIRB alone at an iron concentration of 0.125 µg/µL showed a T2 of 385±33 ms, whereas for muJ591:MIRB-treatment at 0.1 µg/µL, the T2 was 272±18 ms, showing that at a similar iron concentration, we can still differentiate between MIRB uptake and muJ591:MIRB binding by the T2 relaxation time values. DU145 cells treated with MIRB alone at an iron concentration of 0.125 µg/µL had a T2 of 161±18 ms, and this value was consistently faster than DU145 cells treated with muJ591:MIRB. This showed that both cell lines were taking up MIRB when it was not conjugated, and once MIRB was conjugated to antibody, only the PSMA-positive LNCaP cells were detected as changing their T2 relaxation time values. This confirmed that muJ591:MIRB complex detection of PSMA-positive LNCaP cells using a clinical 3T MRI was feasible and sensitive for iron concentrations as low as 0.02 µg/µL (0.035 µg/µL protein concentration).

## Discussion

We were able to stably conjugate the prostate cancer-detecting muJ591 antibody and MIRB to generate antibody:SPIO complexes with an iron to antibody concentration that is sufficient to serve as a functional MR contrast agent detectable by clinical MRI *in vitro*, flow cytometry, and immunofluorescence based methods.

Several studies have shown how antibodies conjugated to super-paramagnetic iron oxide particles were used for specific MR imaging of human lymphocytes, the Her-2/neu receptor in breast cancer cells, and apoptotic cells [20], [25], [27]. These studies used ICP-AES to determine levels of iron to determine loading concentration of the conjugates. However, none of these studies explicitly elaborated on the relative amount of iron per antibody or ligands of interest. In the present study, we used a combination of Bradford protein and ICP-AES based assays to determine the total protein content and iron loading per antibody as an indirect measure of conjugation efficacy. This allowed us to confirm the presence of iron in the complex and to characterize the response as a function of iron concentration, which is valuable information for MR imaging since SPIO dosage has been previously established based on iron concentration and this can be then used to ensure enough contrast sensitivity.

When 1 µg/µL of muIgG antibody or muJ591 antibody were conjugated to MIRB using our two-step carboiimide reaction, we recovered in average 0.45±0.03 µg/µL of muIgG:MIRB and 0.56±0.05 µg/µL of muJ591 antibody:MIRB complex. This protein recovery after the conjugation was estimated by Bradford protein assays performed before and after the reaction.

The MIRB cluster-conjugated muJ591 antibody had similar values of elemental iron per antibody to the muIgG antibody. It is therefore likely that we have similar nanoparticle per antibody ratio and the muIgG is therefore an acceptable control. However, when the T2 relaxation time was obtained, the muIgG:MIRB had a slower T2 which was very closer to the value obtained for the MIRB nanoparticle alone. The conjugation with the muJ591 antibody seems to have an effect on the relaxation time in a favorable direction to its detection since the T2 relaxation time becomes faster.

Note that, due to limitations in sample preparation and the resolution of the SEM instrument which we used, we were not able to accurately estimate the particle size of the conjugates on the SEM images. The drying step performed during the sample preparation step to immobilize the muJ591:MIRB conjugates on to the Nucleopore filters resulted in formation of crystalline buffer salts which are prominently seen in the SEM images as large micrometer-size structures. However, by performing the EDX elemental mapping we were able to confirm the presence of the muJ591:MIRB conjugates immobilized within these structures. We therefore used dynamic light scattering to obtain particle sizes of the conjugates.

When equal protein concentrations of antibody:MIRB conjugates were subjected to NMR analysis, we found that the T2 relaxation time of the muJ591:MIRB was similar to the one for the muIgG:MIRB complex. This could be attributed to the similar amount of elemental iron contents and molecular size of the conjugates. The T2 relaxation time measurements with the MRI showed differences between the muIgG:MIRB and the muJ591:MIRB complexes. The difference between NMR and MRI results can be explained by the use of a standard multi echo spin echo sequence on the MRI and calculations based on processed MR images intensity changes. Nevertheless, our findings showed that the antibody:MIRB complex presents a significantly faster T2 relaxation time that makes it a good candidate as MR contrast agent.

The success of our two-step carboiimide conjugation could be confirmed by determining the changes in peak λ_max_ values as well as the fluorescence intensity of Rhodamine-B fluorophore in the MIRB nanoparticles. The observed Rhodamine-B quenching was exploited to indirectly determine the success of antibody conjugation to MIRB nanoparticles. Fluorescence intensity measurements were performed routinely as quality control assessment measure following each synthesis experiments and to determine the overall loading concentration. Our results agree with previous reports that indirectly suggest that the solvent polarity and proximity of quenching species to the metal-fluorophore conjugation system may play a role in the fluorescence quenching [35].

Our findings suggest that the binding of the conjugate muJ591:MIRB to the PSMA-positive cells is highly specific. The control muIgG:MIRB conjugate could not be detected in the MRI images or T2 relaxation time maps of the cells. We have used a muIgG antibody as an experimental control antibody for both synthesis and validation studies. Several studies used IgG antibody to demonstrate the specificity of their functional MR contrast agents [19]–[25], [27], [36]. This control was used to test for non-specific binding to the tumor cells, while the muJ591:MIRB conjugates would strongly bind to the PSMA expressing cells. In our *in vitro* experiments, the muIgG:MIRB conjugates were easily cleared following the washes during the immunostaining steps while the muJ591:MIRB conjugates were found to remain adherent to the PSMA expressing LNCaP cells. Thus by using muIgG antibody as a control, we ensured the overall efficacy and functionality of the complexes.

We additionally determined potential stability issues while formulating the conjugates in different buffers in order to maximize the functionality and specificity of the antibody:MIRB conjugates for future *in vivo* studies. We first determined the best buffer in which the antibody:MIRB conjugates would maintain stable surface charge and remain in solution without flocculation or aggregation during storage. We performed a zeta potential analysis (data not shown) and observed that the 0.01 M sodium bicarbonate buffer could maintain uniform surface charge of antibody:MIRB complex. Preliminary *in vivo* tests were performed (Lakehead University Animal Care Committee approved protocol 19-08-09) and confirmed 0.01 M sodium bicarbonate as the best buffer for *in vivo* use.

Androgen-dependent LNCaP, which specifically express PSMA receptors when compared to other prostate cancer cell-lines [37]–[41], are extensively used to generate xenograft tumors in athymic nude mice [38], [39]. Hence LNCaP cells were used in our study to test the muJ591:MIRB conjugates. We selected the DU145 prostatic cell line as a negative control, since it is androgen-independent and has a high metastatic potential but it does not express PSA or PSMA receptors [40], [41]. Using immunofluorescene staining, we validated that our muJ591:MIRB antibody complex was able to specifically detect PSMA expressing LNCaP cells *in vitro*. Flow cytometry-based detection showed as well that the muJ591:MIRB was binding to LNCaP cells.

Flow cytometric and immunofluorescence approaches were both successful in discerning the binding capacity and also the relative uptake capacity of muJ591:MIRB conjugates in both DU145 and LNCaP cells. Flow cytometric-based detection was more accurate in determining the extent of binding, which is especially useful when picogram level of contrast agents are used. A future line of experiments will be to determine the antibody:MIRB uptake kinetics using confocal microscopy.

We could detect the presence of muJ591:MIRB on live PSMA positive cells using a clinical 3T MR scanner. The change in T2 relaxation time was significant and standard multi-echo, spin-echo sequences allowed us to quantify it. It should therefore be possible to obtain images using standard T2-weighted imaging at echo times selected by the T2 relaxation time value of the complex, as well as T2-mapping techniques. There is however a limit on the detection capabilities of the technique related to the amount of cells and therefore SPIO signal.

Because prostate cancer cell lines take up non-conjugated MIRB and this also modifies the T2 relaxation time, special caution should be taken when comparing non-conjugated SPIO-based contrast agents to the antibody:SPIO agents. However, the fact that non-conjugated MIRB is taken up by multiple cells and the antibody:MIRB only by specific cell types should give the necessary specificity to detect PSMA-positive cells only.

For live cell *in vitro* detection using the MR scanner we suspended cells on serum-free media during the imaging. Other more tissue-like experimental settings such as agar or agarose were preliminary investigated but they were found to strongly modify the T2 relaxation time and reduce the sensitivity of the detection.

Our findings showed that the muJ591:MIRB treated LNCaP cells showed significantly reduced cell adhesion and increased Annexin V expression, along with significant reduction in cell proliferation. Tumor cells that are less adherent may be more likely to cause metastasis as there is enhanced migratory and invasive potential. At the same time, some studies also show that certain tumor cells that become less adherent may undergo anoikis, a form of apoptosis induced by anchorage-dependent cells detaching from extracellular matrix (ECM) [42], [43]. In fact, if these cells remained adherent, they would resist apoptosis by forming tumor niches that grow uninhibited when the conditions become adverse or to resist chemotherapy [31], [44]. ECM -integrin interaction based signaling is a major molecular signaling pathway that contributes to cancer cell survival and resistance to chemotherapy [42]. It has been postulated that PSMA modulates the integrin signaling pathway that allows prostate cancer cells to resist apoptosis [18], [31]. This implies that muJ591:MIRB can functionally interact with the PSMA receptor possibly by blocking its modulatory effects on integrin receptors. This interaction may help disrupt anti-apoptotic, PSMA-mediated ECM/integrin signal transduction by promoting clathrin-mediated PSMA receptor internalization, which also induces other pro-apoptotic signaling pathways [6], [18], [30], [31]. Hence, we believe that muJ591:MIRB nanoparticles offer potential as a therapeutic agent by neutralizing PSMA's carcinogenic functions.

Knowing that muJ591:MIRB likely has pro-apoptotic effects on prostate cancer cells, care should be taken with the imaging timing to strike a balance between detection of treated cells and their death due to the muJ591. The *in vivo* images should be done at different time points. A longer time course study would be necessary for long term *in vivo* effects targeting PSMA-expressing cells.

Recent years have given rise to the field of theranostics, whereby non-invasive detection and treatment are possible due to advances in nanoparticle synthesis and advanced imaging methods. Theranostic nanomedicines, mostly nanoparticles carrying therapeutic or diagnostic markers in form of targeted small interference RNA or antibodies, have been designed to improve current cancer therapies thus addressing specific limitations of current treatment and diagnostics approaches [44]–[46]. Treatment monitoring is enabled by either attaching different imaging moieties to or exploiting the intrinsic properties of some of these nanoparticles, such as superparamagnetism for MRI. MRI tracking of magnetically labeled cells is non-invasive and cost-efficient, as they are suitable for longitudinal studies [47]. With recent advances in biomarker research, antibody engineering, bioconjugation chemistry, and imaging methods, cancer theranostics allow for specific targeting of biomarkers of tumor progression for detection and subsequent non-invasive treatment [48].

In conclusion, we demonstrated the generation and characterization of muJ591:MIRB nanoparticle conjugates, which can specifically detect PSMA expressing LNCaP cells using 3T Clinical MRI. Our findings suggest that muJ591:MIRB could be a promising diagnostic agent that shows therapeutic effects —a possible prostate cancer theranostic agent. This study also paves the way for our next goal, to appropriately validate the MR contrast agent for *in vivo* prostate cancer MRI studies.
